# The Role of Ferric Nitrilotriacetate in Renal Carcinogenesis and Cell Death: From Animal Models to Clinical Implications

**DOI:** 10.3390/cancers14061495

**Published:** 2022-03-15

**Authors:** Yasumasa Okazaki

**Affiliations:** Department of Pathology and Biological Responses, Graduate School of Medicine, Nagoya University, 65 Tsurumai-cho, Showa-Ku, Nagoya 466-8550, Japan; samasuya@med.nagoya-u.ac.jp; Tel.: +81-52-744-2089; Fax: +81-52-744-2091

**Keywords:** oxidative stress, iron, ferric nitrilotriacetate, lipid peroxidation, renal cell carcinoma, mesothelioma, ferroptosis

## Abstract

**Simple Summary:**

Iron is essential for cellular growth and survival. As a consequence, iron deficiency causes pleiotropic effects on the organism, while iron overload is also deleterious by means of oxidative tissue injury, which causes hepatic cirrhosis, diabetes mellitus, and cardiomyopathy in humans. Non-heme iron comprises ferric ion (Fe(III)), which is much more prominent in the transferrin, ferritin, or labile iron pool than the ferrous ion (Fe(II)); in contrast, ferrous ion yields more reactive oxygen species (ROS) than ferric ion does. In rodents, ferric nitrilotriacetate (Fe-NTA) elicits hepatic and renal oxidized lipids via a glutathione-cycle-dependent iron reduction that eventually causes renal cell carcinoma (RCC). In addition to iron-mediated carcinogenesis, ferroptosis is triggered by the iron-dependent accumulation of lipid peroxidation to lethal levels. Here, the mechanisms of iron- and ROS-mediated RCC and the therapeutic possibility of ferroptosis are discussed.

**Abstract:**

Iron is essential for cellular growth, and various ferroproteins and heme-containing proteins are involved in a myriad of cellular functions, such as DNA synthesis, oxygen transport, and catalytic reactions. As a consequence, iron deficiency causes pleiotropic effects, such as hypochromic microcytic anemia and growth disturbance, while iron overload is also deleterious by oxidative injury. To prevent the generation of iron-mediated reactive oxygen species (ROS), ferritin is synthesized to store excess iron in cells that are consistent with the clinical utility of the serum ferritin concentration to monitor the therapeutic effect of iron-chelation. Among the animal models exploring iron-induced oxidative stress, ferric nitrilotriacetate (Fe-NTA) was shown to initiate hepatic and renal lipid peroxidation and the development of renal cell carcinoma (RCC) after repeated intraperitoneal injections of Fe-NTA. Here, current understanding of Fe-NTA-induced oxidative stress mediated by glutathione-cycle-dependent iron reduction and the molecular mechanisms of renal carcinogenesis are summarized in combination with a summary of the relationship between the pathogenesis of human RCC and iron metabolism. In addition to iron-mediated carcinogenesis, the ferroptosis that is triggered by the iron-dependent accumulation of lipid peroxidation and is implicated in the carcinogenesis is discussed.

## 1. Introduction

Iron is the most abundant transition metal in the body. Iron-containing proteins utilize iron at catalytic sites and facilitate oxygen transport, oxygen metabolism, energy metabolism, DNA synthesis, and repair [1]. Iron deficiency causes impaired growth and cellular functions; thus, mammals develop recycling systems to keep iron in the body. The absorption of dietary iron is tightly regulated at the duodenum through divalent metal transporter 1 (DMT1). Indeed, a previous study showed that *Dmt1*-deficient mice were unable to survive without red blood cell transfusion [2] and that the mutation of human *DMT1* causes hypochromic microcytic anemia. The concentration of chelatable iron within body fluids is normally maintained at a low level (less than 1 μM), while iron overloading conditions elevate the redox-active and chelatable non-transferrin-bound iron concentration to 1–20 μM, causing damage to several tissues by the induced reactive oxygen species (ROS) [3]. Iron-induced ROS exacerbate multiple diseases, such as neurological diseases (Alzheimer’s, Huntington’s, and Parkinson disease’s), cardiovascular system diseases (atherosclerosis by excess lipid peroxidation and hereditary hemochromatosis (HH)-induced cardiomyopathy), hepatocellular carcinoma (HCC) (HH, hepatitis B virus (HBV), or hepatitis C virus (HCV)), or asbestos-related asbestosis, lung cancer, and malignant mesothelioma (MM) [4]. With the current advances that have been made in nutrition, iron deficiency is quite rare, except in women before menopause and in patients who suffer from gastrointestinal bleeding or eating disorders. In this article, investigations of the iron-triggered oxidative stress that causes carcinogenesis or cell death, namely ferroptosis, are reviewed.

## 2. Iron-Induced Renal Carcinogenesis

### 2.1. Ferric Nitrilotriacetate (Fe-NTA) Induces Renal Oxidative Injury and Eventually Leads to Renal Cell Carcinoma (RCC)

Animal models of Fe-NTA injections were first developed as an experimental iron overload model that caused diabetes mellitus by means of iron loading to pancreatic endocrine cells [5]. The use of NTA enables ferric ions to be maintained in a mono- or oligo-state in solution at neutral pH by forming a weak iron complex [6] and a short-lived intermediate complex of NTA-ferric ion-transferrin (Tf) [7]. The peak iron concentration in serum after a single intraperitoneal injection of 10 mgFe/kg body weight was observed at 1 h and returned to untreated levels after 24 h [6]. A single Fe-NTA injection also elevated hepatic lipid peroxidation and the release of serum hepatic injury markers, such as aspartate aminotransferase (AST) and alanine aminotransferase (ALT) [6]. In 1982, RCC development was observed in male Wistar rats after repeated injections of Fe-NTA [8]. Oral administration of Fe-NTA did not induce RCC [8], which is consistent with the tight regulation of intestinal iron absorption [9]. Despite the high incidence and early development of Fe-NTA-induced RCC, HCC is rare in wild-type (Wt) littermates of rats and mice [8]. Indeed, the combination of a single intraperitoneal injection of *N*-diethylnitrosamine (DEN) (100 mg/kg body weight), which is used for the initiator of HCC development [10], and repeated intraperitoneal injections of Fe-NTA elevated the incidence of RCC, while no HCC was developed with the injection of DEN alone or with the combined injections of DEN and Fe-NTA in male ddY mice [11] or male Wistar rats [12], suggesting that the liver is more robust against Fe-NTA-induced oxidative stress than the kidney.

Repeated intraperitoneal injections of copper-NTA, which induced hepatic cirrhosis, hemolytic anemia, and renal tubular necrosis [13], led to a higher incidence of RCC (12/32) than HCC (1/32) and hepatic sarcoma (1/32), while these cancers developed more slowly than Fe-NTA-induced RCC did in male Wistar rats [14]. In another study, 100% (13/13) of the Long–Evans Cinnamon (LEC) rats, which harbor an *Atp7b* mutation, developed necrotizing hepatitis in addition to developing HCC (13/13) and RCC (4/13) at 111–120 weeks [15]. These HCC and renal tubular injuries were attenuated by a copper-chelating drug, *D*-penicillamine [15], indicating that copper is critical for these diseases, while Wilson’s disease patients, who carry *ATP7B* mutations, with increased copper accumulations and ROS in the mitochondria develop neurological, psychiatric, ophthalmological, and hepatic symptoms without frequent renal involvement [16]. On the other hand, aluminum-NTA, which causes acute tubular necrosis and regenerative epithelial cells, did not induce RCC in rats [17].

Among the metal chelators, NTA initiated lipid peroxidation more efficiently than diethylenetriaminepentaacetate (DTPA), ethylenediamine-N, N, N′, N′-tetraacetate (EDTA), ADP, and deferoxamine (DFO) did when chelated with iron [18]. The standard redox potentials range from +0.59 V for Fe-NTA [19], +0.03 V for Fe-DTPA, +0.11 V for ionic form of iron, +0.12 V for Fe-EDTA, +0.10 V for Fe-ADP, −0.45 V for Fe-DFO, to −0.40 V for Fe-Tf at pH 7.0 [20], indicating the highly oxidizing potency of Fe-NTA. Indeed, the intraperitoneal injection of Fe-NTA elevated the renal 2-thiobarbituric acid reactive substances (TBARS) more than when the same ferric chloride dosage without a chelator was applied, while Fe-EDTA or Fe-DFO did not induce a significant increase in the renal TBARS in male Wistar rats [21]. On the other hand, EDTA, DTPA, and citrate with iron (molar ratio 1:1) elicited a catalytic reduction of H_2_O_2_ that disappeared when these chelators were present at concentrations that were over a hundred times greater than the iron concentration [22], indicating that the substrate or ferric–chelator complex ratio are important in such assessments. In addition to NTA, ethylenediamine-N,N′-diacetate (EDDA) with ferric ion induced renal lipid peroxidation and RCC [23], and Fe-iminodiacetate (IDA) also elicited renal lipid peroxidation and proximal tubular necrosis, while N-(2-hydroxyethyl)iminodiacetate (HIDA), ethylenediamine-N,N′-dipropionate (EDDP), and N,N′-bis(2-hydroxybenzyl)ethylendiamine–N,N′-diacetate (HBED) with ferric ions were ineffective in initiating renal lipid peroxidation in male Wistar rats [24]. While in sera, the iron concentration was not significantly different between the initiating lipid peroxidation group (Fe-NTA and Fe-EDDA pH 7.4; and Fe-IDA pH 5.2, 6.2, and 7.2) and the absence of the lipid peroxidation group (Fe-EDTA pH 7.4 and Fe-IDA pH 8.2) in Wistar rats, indicating the importance of the iron–chelator complex as an origin of ROS [24]. The origin of different iron chelate complexes in ROS generation is hypothesized as being the result of Fe-NTA and Fe-IDA forming a μ-oxo dimer iron at a neutral pH by means of crystal X-ray analysis [24,25].

### 2.2. Mechanisms of Fe-NTA-Induced Renal Oxidative Injury

**Figure 1:**
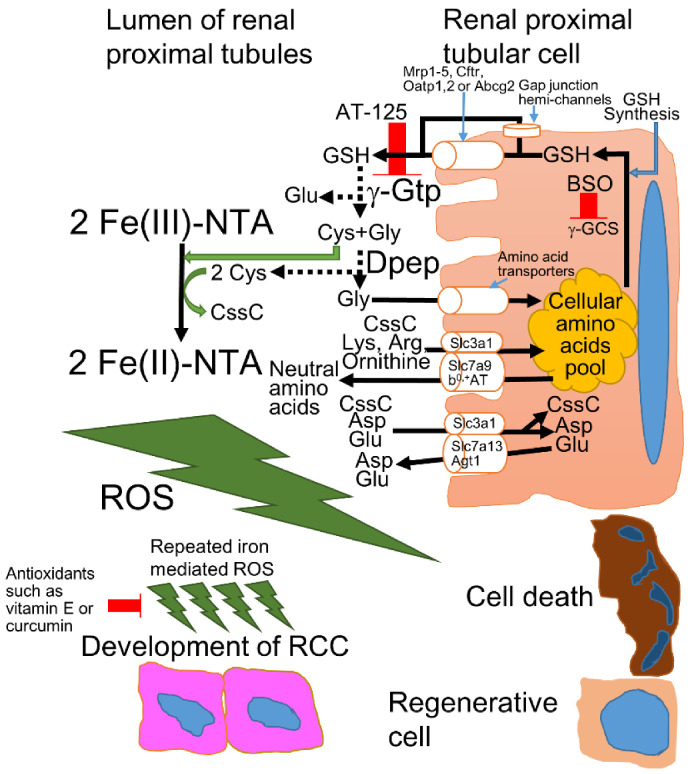
The mechanism of Fe-NTA-induced oxidative injury in the renal proximal tubules of rodents. Approximately 95–99% of all amino acids are reabsorbed in the proximal convoluted tubule and proximal straight tubule; thus, with very few exceptions, individual amino acids are transported by more than one transporter to provide backup capacity for absorption [34]. The intracellular GSH is exported from the cytoplasm to the lumen through GSH exporters, such as multidrug resistance-associated protein (Mrp) 1–5, cystic fibrosis transmembrane regulator (Cftr), organic anion transporting polypeptide (Oatp) 1 and 2, ATP-binding cassette superfamily G2 (Abcg2), or gap junction hemi-channels [27]. GSH is degraded by membrane-associated enzymes, such as γ-glutamyl transpeptidase (γ-Gtp) [8,27]. The Cys-Gly can be degraded by membrane-bound dipeptidases (Dpep) [8,27] or imported to the cytoplasm directly by amino acid transporters [27,34]. Among the constituents of GSH, Gly is transported by many amino acids transporters [34], while Cys reduces Fe(III)-NTA to yield Fe(II)-NTA and cystine (CssC), triggering iron-dependent oxidative injury and carcinogenesis in the renal proximal tubules [8]. The CssC is transported into the cytoplasm via the related b^0^ amino acid transporter/b^0,+^ amino acid transporter (rBAT/b^0,+^AT), which is also called the Slc3a1/Slc7a9 complex, with anionic amino acids (Lys and Arg) and ornithine [34,35] or rBAT with Slc7a13, also known as aspartate/glutamate transporter 1 (Agt1), with cationic amino acids (Asp and Glu) [36]. In physiological conditions, more than 90% of CssC is reabsorbed via rBAT/b^0,+^AT, and the residual CssC is reabsorbed by rBAT/AGT1 in the renal proximal tubules [36].

After the intraperitoneal injection of Fe-NTA, Fe-NTA is delivered to renal proximal tubules through systemic circulation and glomerular filtration. At the renal proximal tubular lumen, Fe-NTA is reduced to ferrous-NTA by cysteine or cysteinylglycine, which are yielded by the glutathione (GSH) cycle (Figure 1). Although GSH has a redox potential similar to that of N-acetyl-cysteine (NAC) [20], GSH does not trigger the reduction of Fe-NTA at the same efficiency as cysteine or cysteinylglycine [26]. In brush border membrane, γ-glutamyl transpeptidase (γ-GTP)-mediated GSH cycling is presumed to maximize the re-absorption of filtrated plasma GSH based on the fact that the enzymatic activity of γ-GTP is the highest in the renal proximal tubular cells and the inhibitor or genetic impairment of γ-GTP increased the urinary excretion of GSH [27]. Pretreatment with AT-125, which inhibits γ-GTP, or butionine sulfoximine (BSO), which inhibits γ-glutamylcysteine synthetase (γ-GCS), attenuates Fe-NTA-induced renal lipid peroxidation, which explains why the distal tubules are not damaged severely by Fe-NTA [28]. The mechanism of Fe-NTA-induced oxidative injury is hypothesized to form a binuclear Fe-O-Fe unit structure (μ-oxo dimer iron), which oxidizes the surrounding biomolecules [29]. Indeed, the modification of the glutamate α-COO^−^ ligand in GSH allowed the reduction of Fe-NTA and the elevation of lipid peroxidation in rat liver microsomes, suggesting the iron reducing capacity of GSH depends on liganding more than the redox potential and thiol pK_a_ [26], and thiolate-ligated iron(II) sites have been shown to be essential for the function of many metalloenzymes [30], indicating the importance of μ-oxo dimer iron during redox reactions. Notably, Fe-NTA generated alkyl and peroxyl radicals in the renal tissue in an electron paramagnetic resonance (EPR) study [31]; thus, a ferrous-NTA-catalyzed Fenton reaction is presumed to trigger renal carcinogenesis [32]. These results indicate the importance of the GSH cycle on the brush border membrane in the renal proximal tubules. In addition to these catalytic reactions, γ-GTP was overexpressed in hepatic preneoplastic lesions in rats who had been treated with chemical carcinogens, indicating the tumor promotional role of γ-GTP by allowing the salvaging of extracellular GSH [33].

The castrated male mice and female mice all survived, while all of the male mice died from repeated Fe-NTA injections in the A/J strain, indicating the sex differences against Fe-NTA-induced oxidative stress [37]. Indeed, the administration of testosterone in castrated male mice and female mice elevated renal lipid peroxidation to levels that were as high as those seen in male mice after Fe-NTA injection [37]. In contrast, estriol attenuated Fe-NTA-induced renal lipid peroxidation in male ddY mice [38]. Furthermore, castration and estradiol suppressed Fe-NTA-induced renal carcinogenesis; conversely, ovariectomy and testosterone increased the frequency of renal carcinogenesis in Wistar rats [39]. Furthermore, the half-life of GSH in males was shorter than that observed in females, something that can be partially explained by the increased γ-GTP activity in males [40,41]. These renal and hepatic GSH cycles were delayed by castration and estradiol treatments in male mice, while testosterone accelerated the cycles in female C57BL/6 mice [40]. However, the intravenous injection of estrogen 5 min prior to intraperitoneal Fe-NTA injection was ineffective in protecting male mice, indicating that estradiol did not attenuate Fe-NTA by direct chelating or scavenging ROS [38]. These results indicate that feminization is critical to protect the liver and kidney from Fe-NTA-induced oxidative injury via an altered GSH cycle and/or feminized protein expression.

Recently, the male-specific expression of the cystine transporter Slc7a13 was observed in a murine kidney model and was shown to potentially be able to modulate the renal GSH cycle, although perfect palindrome estrogen- and androgen-responsive elements were not detected in *Slc7a13* [36]. Among the various cystine transporters, Slc3a1 forms a heterodimer for the amino acid transport system and showed its highest expression in the non-neoplastic kidney and intestine [42]. Further, SLC7A11, which forms a heterodimer with SLC3A2 and is known as a xCT (cystine/glutamate antiporter), has higher expression in RCC than in the non-neoplastic kidney and has been shown to be related to a worse prognosis in RCC [43]. The loss of Slc7a11-dependent cystine uptake increased the susceptibility to ROS in *Slc7a11*-deleted mice, while renal stones, which are frequently caused by cystinuria, were not seen [44], suggesting xCT is not critical in reabsorption of filtrated cystine. These results are consistent with previous results indicating that cystinuria is caused by the mutation of either *SLC3A1* or *SLC7A9* [34] (Figure 1). Furthermore, the kidney-specific ablation of the murine androgen receptor (AR) caused increased ammonia excretion and increased expression of the Na^+^-K^+^-2Cl^−^ cotransporter (NKCC2) and phosphoenolpyruvate carboxykinase (PEPCK), while it triggered the suppression of renal size, Na^+^/H^+^ exchanger isoform 3 (NHE3), and Na^+^-bicarbonate cotransporter 1 (NBCe1) [45]. Future studies may reveal feminization-induced protection against iron-induced oxidative stress.

### 2.3. Molecular Mechanisms of Fe-NTA-Induced Carcinogenesis in Rodents Compared to Human RCC

The *von Hippel*–*Lindau* (*VHL*) mutation shows that it is able to increase susceptibility to hemangioblastoma in the retina or central nervous system, neuroendocrine tumors (notably pheochromocytoma), and familial or sporadic clear-cell RCC (ccRCC) at a high frequency in humans [46]. This *Vhl* mutation was not detected in Fe-NTA-induced RCCs [47,48]. Furthermore, no *H*-, *K*-, or *N-ras* gene mutations (codon 12, 13, or 61) were detected via direct sequencing in the 12 Fe-NTA-induced RCCs [49] and in the 16 RCCs [50] with low incidences of *p53* mutations, showing rates of (1/12) and (2/16), respectively. While low mutation frequencies of *Vhl*, *ras*, and *p53* were detected in the Fe-NTA-induced RCC model, the combined deletion of *Vhl*, *p53*, and *Rb1* in the renal tubular cells caused ccRCC in mice [51]. The single or double conditional knock out (cKO) of *Vhl* and/or *p53* and *Rb1* in the renal tubular cells showed only the development of cysts and microscopic neoplasms, indicating the importance of the simultaneous disruption of the p53 and G1/S cell cycle checkpoint [51,52]. Meanwhile, the double cKO of *Vhl* and *BRCA1-associated protein 1* (*Bap1*) developed ccRCC faster than the double cKO of *Vhl* and *polybromo 1* (*Pbrm1*), which encodes the BRG1-associated factor 180 (BAF180), a component of the SWI/SNF-B chromatin-remodeling complex [53]. The absence of RCC development by means of murine single *Vhl* disruption is presumed to be due to the fact that murine *Vhl* is located on chromosome (chr) 6, whereas *Bap1* and *Pbrm1* are located on chr 14, causing the avoidance of the simultaneous loss of *Vhl* and *Bap1* or *Pbrm1*, while *BAP1*, *PBRM1*, and *VHL* are located on chr 3p21 and 3p25 and are frequently co-deleted in ccRCC [53].

**Figure 2:**
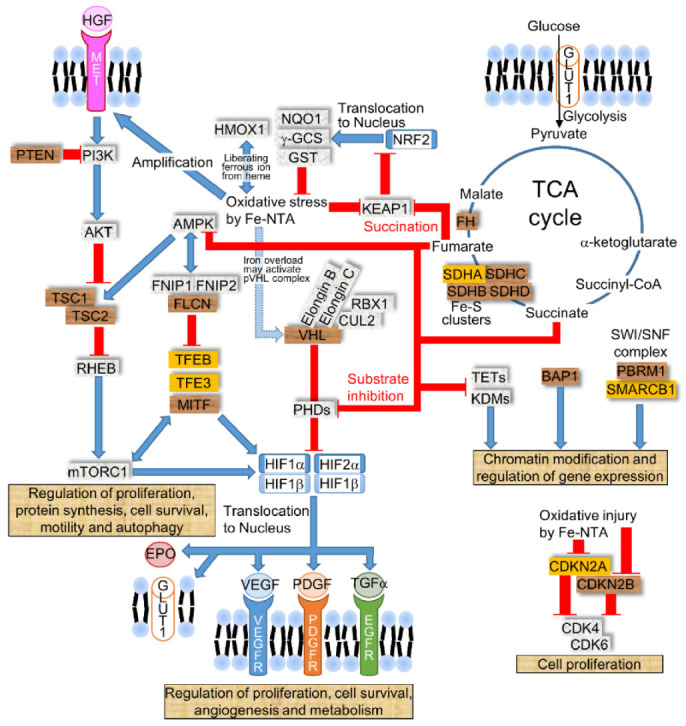
The dysregulated signaling pathways in RCC. RCC is caused by several signaling pathways, such as HIF, MET kinase, mTOR, TCA cycle, cell cycle regulation, and chromatin modification and regulation. Brown tags represent a gene that causes RCC via germline or somatic alterations, such as *VHL* (*von Hippel–Lindau*), *BAP1* (*BRCA1-associated protein 1*), *PBRM1* (*polybromo 1*), *MET*, *PTEN* (*phosphatase and tensin homolog*), *TSC1/2* (*tuberous sclerosis 1/2*), *FLCN* (*folliculin*), *MiTF* (*microphthalmia-associated transcription factor*), *SDHB/C/D* (*succinate dehydrogenase B/C/D*), *FH* (*fumarate hydratase*), and *CDKN2B* (*cyclin-dependent kinase inhibitor 2B*). Orange tags represent a gene that is frequently mutated in sporadic RCCs, such as *SDHA*, *TFEB* (*transcriptional factor EB*), *TFE3* (*transcriptional factor E3*), *SMARCB1*, and *CDKN2A*. The blue arrow indicates the activation pathway, and the red line indicates signal transduction suppression. Acronyms: PHDs (prolyl hydroxylases), HGF (hepatocyte growth factor), EPO (erythropoietin), AMPK (AMP-activated protein kinase), FNIP1/2 (folliculin-interacting protein 1/2), RHEB (ras-homolog expressed in brain), VEGF (vascular endothelial growth factor), PDGF (platelet-derived growth factor), TETs (ten-eleven translocations), and KDMs (lysine-specific demethylases).

The VHL complex, which comprises pVHL, elongin B, elongin C, Cullin-2, and RBX1, degrades hypoxia-inducible factor (HIF) 1α and 2α by ubiquitination once the HIFs are hydroxylated by the α-ketoglutarate-dependent prolyl hydroxylases (PHDs) (Figure 2). In *VHL*-mutated ccRCCs, both HIF1α and HIF2α are important, due to the frequent *HIF1A* deletions (40–55%); furthermore, a murine model demonstrated the sufficient inhibition of ccRCC pathogenesis by Hif2α degradation and the necessity for the ccRCC formation by Hif2α stabilization [46]. More recently, the inhibition of the HIF2α and HIF1β heterodimer by therapeutic small molecules has been shown to effectively suppress ccRCC progression in patients [54].

The tuberous sclerosis complex (TSC) is caused by a germline loss-of-function mutations of either the *TSC1* or *TSC2* gene develops a wide range of tumors in the brain (subependymal giant cell astrocytoma and cerebral cortical tuber), heart (rhabdomyoma), kidney (angiomyolipoma, cyst, and RCC), lung (lymphangioleiomyomatosis), or skin (angiofibroma) [55]. In a hereditary RCC Eker rat model, *Tsc2* was shown to be the causative gene; however, the *Tsc2* mutation was only detected in 3% (1/34) of Fe-NTA-induced RCC [48]. On the other hand, biallelic *Tsc2* mutations were observed in N-ethyl-N-hydroxyethylnitrosamine (EHEN) with KBrO_3_-induced RCCs (2/3) and DEN-induced RCCs (3/5) [56]. These results indicate that different renal carcinogens, which generate ROS, confer selective genes. In *Tsc2*-mutated Eker rats, the homozygous deletion (HD) of *Cdkn2a* was detected in the cell lines (5/7 or 14/24) and was not detected in the primary RCCs [57,58]. These results suggest that the loss of *Cdkn2a* plays a tumor progressive role in vitro in *Tsc2*-mutated RCC [58]. Meanwhile, in Fe-NTA-induced RCC, the methylation of the promoter region or the loss of heterozygosity (LOH), or HD was detected in *Cdkn2a* (21/39) and *Cdkn2b* (12/39), respectively [59]. The allelic loss of *Cdkn2a* was detected by fluorescent in situ hybridization (FISH) in male Wistar rats after receiving Fe-NTA injections for 3 weeks [60]. These results suggest that the loss of *Cdkn2a/2b* is triggered at the early tumor initiative stage. Indeed, germline mutations in *CDKN2B* predisposed to ccRCC, while the copy number abnormalities in the *CDKN2A/2B* region were detected in approximately 15% of sporadic ccRCC cases [61], indicating the presence of a common carcinogenic pathway in rodents and human RCC. In addition to the inactivation of *Cdkn2a/2b*, *Met* amplification was significantly associated with increased tumor size in Fe-NTA-induced RCC (Figure 2). Furthermore, transforming growth factor α (Tgf-α), which is induced by Hif and activates epidermal growth factor receptor (Egfr) signaling, was expressed in Fe-NTA-induced rat RCC [62]. While male Wt rats developed Fe-NTA-induced RCC, nearly 90% of Wistar strain or Brown Norway/Fischer344 (BN/F344) F1 hybrid rats during a two-year observation period [32], male Wt mice developed RCC in the A/J strain (18/29), C57BL/6 strain (1/14) [63], and ddY strain (7/20) [11], indicating the difference in the genomic instability against Fe-NTA-induced oxidative injury between the mouse strains and rats [64].

Fe-NTA-induced renal oxidative stress induced the activation of nuclear factor erythroid 2-related factor 2 (Nrf2), which elevated the antioxidative phase II metabolizing enzymes, such as GSH-S-transferases (Gst), NADPH quinone oxidoreductase 1 (Nqo1), heme oxygenase-1 (Hmox1), and γ-Gcs [65,66] (Figure 2). While Hmox1 protects cells from ROS, the release of ferrous ions from heme may elevate lipid peroxidation and cellular injury; thus, the role of Hmox1 in the iron overloaded condition requires further investigation. Not limited to ROS, Nrf2 is also activated by several electrophiles, such as fumarate or curcumin. Curcumin induced phase II metabolizing enzymes [67] as well as attenuated Fe-NTA-induced ROS by direct scavenging that suppress renal carcinogenesis in male ddY mice [11,68]. In addition to curcumin, dietary vitamin E prevented Fe-NTA-induced lipid peroxidation [69] and significantly suppressed Fe-NTA-induced renal carcinogenesis in male Wistar rats [70] (Figure 1). Meanwhile, phlebotomy did not suppress the incidence of Fe-NTA-induced RCC in male Wistar rats [71]. In systemic *Dmt1-IRE* (*iron-responsive element*) transgenic mice, the Fe-NTA-induced renal damage was not obviously elevated [72]. Future studies may reveal the role of iron-induced oxidative damage in renal carcinogenesis that could contribute to identifying chemopreventive compounds for RCC.

### 2.4. Iron Modulates the Expressions of HIF-α by PHDs and HIF2α by IRE/Iron Regulatory Protein (IRP)

**Figure 3:**
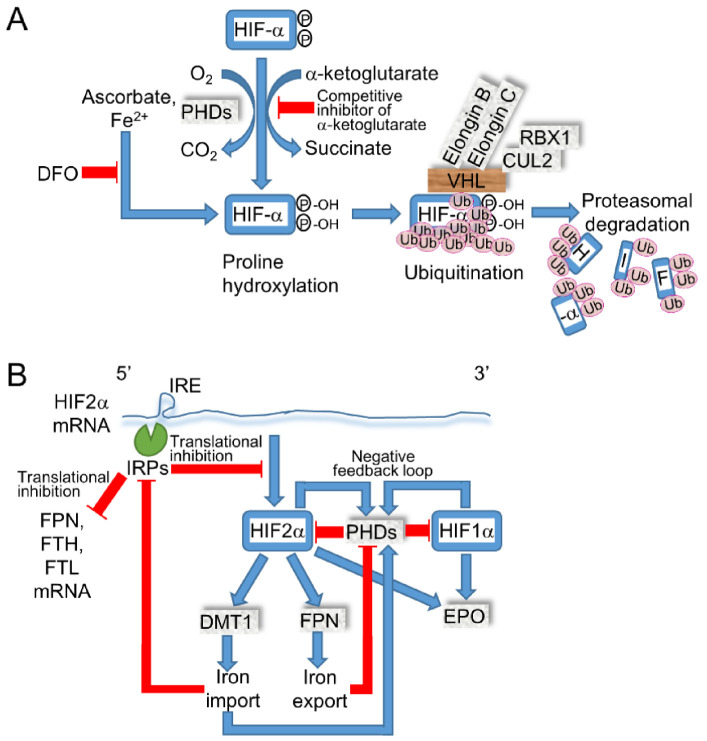
Iron-regulated HIF-α expression. (**A**) HIF-α is hydroxylated by PHDs, which are suppressed by molecular oxygen depletion or iron chelation. The hydroxylations at the proline in HIF-α induce recognition by the VHL complex, which initiates ubiquitination-dependent proteasomal degradation. Competitive inhibitors of α-ketoglutarate are prescribed for renal anemia to inhibit PHDs, and, in turn, stabilize HIF-α [75]. (**B**) HIF2α, which is translationally suppressed by IRE/IRPs as FPN (ferroportin), FTH (ferritin heavy chain), and FTL (ferritin light chain), transcribes DMT1 and FPN [74]. The increased cytoplasmic iron may activate PHDs, while IRE/IRP-dependent translational inhibition is decreased.

The dietary iron efflux into systemic circulation is transported by ferroportin (FPN, SLC40A1), which is internalized and degraded by hepcidin when excess iron or inflammation are sensed in the liver, which regulates systemic iron storage [1]. The translation of *HIF2α* mRNA is regulated by IRE/iron regulatory protein 1 or 2 (IRP1/2) through IRE in the 5′ untranslated region (UTR), while *HIF1α* does not contain IRE in mRNA. Thus, iron deficiency elevated the protein expression level of HIF1α and 2α via suppressed PHDs; in contrast, HIF2α was decreased by elevated IRE/IRP activity [46] (Figure 3). In murine embryonal fibroblasts (MEF) from *Irp1* KO mice, the Hif2α protein was expressed in both iron-sufficient and DFO-treated conditions and was higher than expression levels in the Wt littermates, indicating that Irp1/2 and Phds regulate Hif2α protein expression separately [73]. The intestine-specific ablation of *Hif2α* decreased the mRNA levels of *Dmt1*(*IRE*) and *Fpn* in the duodenum, *hepcidin* in the liver, and iron concentration in serum and the liver [74]. Conversely, the pharmacological inhibition of PHDs, which are clinically prescribed for renal anemia to activate HIF signaling, increased erythropoietin and DMT1, while PHD expression was induced by the HIFs to form a negative feedback loop to prevent the persistent activation of HIF signaling [75]. In addition to DMT1, ZRT/IRT-like protein (ZIP) 14 exhibited iron uptake, while the intestine-specific ablation of *Dmt1* caused a lethal iron-deficiency that was restored by the intraperitoneal injection of iron, indicating that Dmt1 is essential for intestinal iron absorption [76]. The loss of IRE/IRPs-dependent translational suppression increases the levels of protein expression in FPN, the ferritin heavy chain (FTH), and the ferritin light chain (FTL) that have IRE in the 5′ UTR region of the mRNA and decreases intracellular labile iron by exporting iron to outside of the cell or by storing it in proteins to reduce iron-induced cellular injury. When *HIF2α* is mutated to be stabilized by inhibiting the hydroxylation of proline and the subsequent binding to VHL, multiple paraganglioma, duodenal somatostatinoma, and polycythemia are developed [77]. Furthermore, a gain of function in *HIF2α* developed vascular malformation in patients and mouse models that may have been caused by the failure of elevated oxygen tension-induced normal venous regression [78]. These results demonstrate that the continuous activation of HIF2α signaling is not associated with the iron overload phenotype; thus, further study may reveal the interaction of iron and HIF signaling in RCCs. Based on the evidences of iron metabolism, the clinical trial of iron-chelating therapy by DFO is effective in hematological neoplasms that are relatively addicted to iron supply for tumor growth. Furthermore, the clinical trial of DFO for cancer therapy demonstrated partial response in HCC, while only a 7% overall response rate (ORR, the percentage of patients with a predefined reduction in tumor size for a certain duration) was observed in metastatic RCC [1], which may have been caused by the increased HIF-α in the absence of active PHDs. From the viewpoint of iron-mediated PHDs activation, elevated iron in RCC may be useful for a therapeutic modality.

### 2.5. Disruption in Succinate Dehydrogenase (SDH) or Fumarate Hydratase (FH) Causes RCC by Inhibiting PHDs

In addition to *VHL*, which causes familial RCC, the mutation of *SDH* predisposes one to oncocytic RCC and gastrointestinal stromal tumors (GIST) with a lower frequency than paraganglioma and phenochromocytoma. SDH, which oxidizes succinate to fumarate with the reduction of FAD^+^ to FADH_2_ as complex II in the mitochondrial respiratory chain, is assembled by SDHA, SDHB, SDHC, and SDHD subunits [79]. Among the SDH subunits, *SDHB* is mutated most frequently, followed by *SDHC* and *SDHD*, in hereditary *SDH*-deficient RCC, while germline mutations in *SDHA* have been described to cause neurodegenerative diseases, such as Leigh syndrome without neoplastic disorder [80]. In addition to congenital disease, the acquired *SDHA* mutation caused oncocytic RCCs without *SDHB/C/D* mutations [81]. Among *SDHB* mutations, Fe-S cluster mutations in the L(I)YR motif amino acid residues were occupied for 50% of the *SDHB*-mutated RCC and GIST, suggesting the importance of the L(I)YR motif in renal cell carcinogenesis [79]. In addition to SDH, the germline inactivation of *FH* that catalyzes fumarate to malate in the TCA cycle and suppresses AMP-activated protein kinase (AMPK) signaling increases the susceptibility to papillary RCC (type 2) and leiomyomatosis in the skin and uterus [73]. The elevation of succinate or fumarate caused by a metabolic shift in the TCA cycle inhibits the PHDs that activate HIF signaling [82]. In *SDH*- or *FH*-deficient RCCs, the protein expression of HIF1α but not HIF2α was more predominant than ccRCC expression [73,79].

### 2.6. Ischemia Reperfusion and Iron-Mediated ROS Induces Renal Medullary Carcinoma

Sickle cell nephropathy, which causes iron overload in the kidney, is characterized by impaired urinary concentration ability, proteinuria, hematuria, chronic tubulointerstitial nephritis, hemolysis, and increased susceptibility to acute kidney injury (AKI), urinary tract infections, and medullary carcinoma due to altered renal hemodynamics, such as the perfusion paradox, which includes medullary hypoperfusion and renal and/or cortical hyperperfusion, and unique ischemia–reperfusion injury (IRI) with repeated multifocal and microvascular occlusions with subsequent infarction [83]. Renal medullary carcinoma (RMC), which was originally described in 1995 and comprises less than 0.5% of all RCCs, is predominantly detected in young African Americans with the sickle cell trait or sickle hemoglobinopathies [84]. RMC is characterized by a near complete loss of SMARCB1 expression, which is a component of the SWI/SNF chromatin remodeling complex and harbors fewer genetic alterations than other RCCs (cc, ch, and papillary). No *VHL*, *PBRM1*, *BAP1*, *TP53*, or *CDKN2A* mutations have been reported [85], indicating that IRI and iron overload-induced ROS confer *SMARCB1*.

### 2.7. Histopathology of RCC

The histopathologies of ccRCC (VHL disease or BAP1-associated tumor predisposition syndrome), *SDH*-mutated oncocytic RCC, and hereditary papillary RCC (constitutive activation of *MET* (type 1) or *FH*-mutated hereditary leiomyomatosis and RCC (type 2)) represents a single predominant histology, while *TSC*-mutated RCC represents heterogeneous components that show a striking contrast (Table 1) [55,86]. In human chromophobe (ch) RCC, which is caused by the germline mutation of *Tsc1/2* or *FLCN* or *PTEN*, the most frequent mutations were detected in *p53* and *PTEN*, followed by a loss of expression in *CDKN2A* [87]. In addition to these established associations of causative genes with characteristic histopathology, the mutation of the *microphthalmia-associated transcription factor* (*MiTF*), which consists of the basic helix–loop–helix family of transcription factors with TFE3 and TFEB, was shown to cause familial renal cancer and cutaneous melanoma [88]. These results suggest that chromophobe histopathology favors the activation of the mTOR pathway (Table 1).

### 2.8. Iron-Induced Peritoneal Malignant Mesothelioma (MM) in Rats

In addition to RCCs, the repeated intraperitoneal injection of Fe-NTA causes iron deposition on the surface that is susceptible to peritoneal MM development. Indeed, the iron saccharate that accumulated on the serosal surface caused MM development in peritoneum and tunica vaginalis without RCC and pleural MM in male Wistar rats. This mesothelial carcinogenesis was accelerated by simultaneous NTA treatment [90], indicating the importance of elevated chelated and/or mobile iron in the local peritoneal fluid by NTA. The development of MM via the intraperitoneal injection of iron saccharate was predominantly observed in male (16/24) but not female (1/30) BN/F344 F1 hybrid rats [91]. Furthermore, the HD of *Cdkn2a/2b* was detected in iron-saccharate-induced sarcomatoid mesothelioma (SM) (4/5) and epithelioid mesothelioma (EM) (0/6) in BN/F344 F1 hybrid rats [91], and EM (1/5) was observed in male Wistar rats [92]. In three Fe-NTA-induced peritoneal MM, no *ras*, *p53*, *Vhl*, or *Tsc2* mutations were detected [48,49], while in human MM, the most frequent genetic mutations were observed in *CDKN2A/2B*, *NF2* (*neurofibromatosis 2*), and *BAP1* [4,93]. Epidemiological studies indicate that the onset of MM is associated with the exposure of asbestos fibers. Among them, crocidolite and amosite, which contain higher iron in the fiber are more carcinogenic than chrysotile, suggesting that iron accelerates mesothelial injury and carcinogenesis via ROS production [4]. In a rat model of intraperitoneal injection of crocidolite, iron reductions by an iron chelator, deferasirox [94], and by phlebotomy [95] prevented the MM developments, indicating the importance of iron storage in the body. Indeed, phlebotomy is useful in hereditary hemochromatosis patients as well as in HCV-infected patients, as it can suppress hepatitis activity and HCC development [96]. Meanwhile, MM cells that harbor *BAP1* or *NF2* mutations are vulnerable to iron-dependent cell death [4,93].

## 3. Ferroptosis

### 3.1. Concept of Ferroptosis

**Figure 4:**
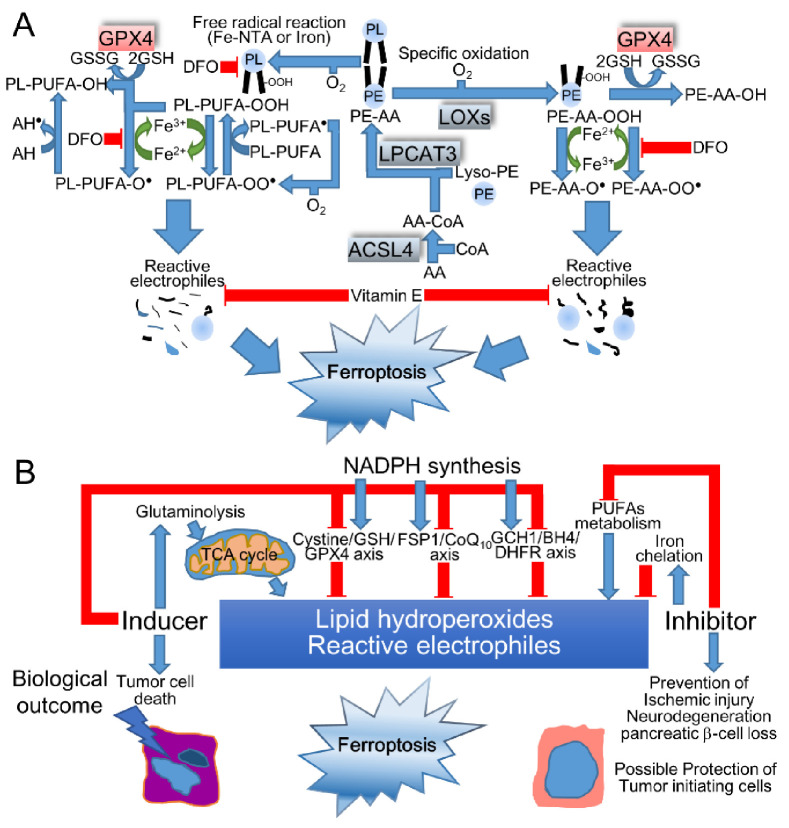
Ferroptotic signaling pathway. (**A**) Among phospholipids (PLs), PE-esterified AA or AdA are predominantly located in the inner leaflet of the plasma membrane or nonbilayer arrangements in contrast to the high PC confinement on the outer membrane or ordered bilayer organization of PC-PUFAs [103]. The lipid hydroperoxides (PL-PUFA-OOH or PE-AA-OOH) are detoxified to alcohol (PL-PUFA-OH or PE-AA-OH) by GPX4. On the other hand, PL-PUFA-OOH and PE-AA-OOH yield iron-mediated peroxyl (R-OO^•^) or alkoxyl (R-O^•^) radicals; finally, reactive electrophiles transduce lipid death signals. These ferroptotic signals are attenuated by suppressing lipid peroxidation with vitamin E or iron chelation with DFO while being enhanced by lipoxygenases (LOXs) or acyl-CoA synthetase long chain family member 4 (ACSL4) or lysophosphatidylcholine acyltransferase 3 (LPCAT3), which mediate PE esterification with PUFAs in addition to PC and phosphatidylserine (PS). The ferroptosis inhibitors are shown in red, while the ferroptosis inducers are shown in blue. (**B**) The compounds that trigger glutaminolysis or that inhibit the cystine/GSH/GPX4 axis, FSP1/CoQ_10_/α-Toc (ferroptosis suppressor protein 1/coenzymeQ_10_) axis, GCH1/BH4/DHFR (guanosine triphosphate cyclohydrolase 1/tetrahydrobiopterin/dihydrofolate reductase) axis, or NADPH synthesis are categorized as ferroptosis inducers that may initiate tumor cell death, while PUFA metabolism suppressors or iron chelators are designated as ferroptosis inhibitors that may confer the prevention of ischemic injury, neurodegeneration, or pancreatic β-cell loss. In addition to these beneficial roles of ferroptosis, the protection of tumor-initiating cells is also speculated.

Ferroptosis is established to dictate the nonapoptotic cell death that is triggered by the iron-dependent accumulation of oxidized lipids, including polyunsaturated fatty acids (PUFAs) to lethal levels [97], and is implicated in the pathogenesis of neurodegenerative diseases (Alzheimer’s, Huntington’s, and Parkinson’s diseases), brain attacks (stroke, intracerebral hemorrhage, and traumatic brain injury), and IRI [98]. Ferroptosis was not originally affected in the necroptotic signaling pathway by receptor-interacting protein kinases (RIPK) 1 and 3, while ferroptosis shares a typical morphology with necrosis, along with small mitochondria, a decreased crista, a condensed inner membrane, and a ruptured outer membrane [99]. Indeed, iron triggers the phospholipids (PL)-PUFA-OOH (hydroperoxide), -OO^•^ (peroxyl radical), and -O^•^ (alkoxyl radical), which are scavenged by α-tocopherol (α-Toc) to form α-Toc^•^ (Figure 4A); thus, long-term iron overload is presumed to be a soil for carcinogenesis by acquiring ferroptosis-resistance [4]. Indeed, PL-PUFA-OOH were significantly elevated in the red blood cells of Alzheimer’s disease patients [100], suggesting that the systemic accumulation of lipid hydroperoxides accelerates cerebral damage. In addition to these pathogenic involvements of these diseases, ferroptosis is an eliminator of potent tumor-initiating cells and tumor cells, indicating a promising therapeutic approach for cancers. When considering the induction of ferroptosis in cancer therapy, ferroptosis increases the risk of neuronal cell death in the central nervous system, potentially aggravating neurological symptoms [101]. Indeed, iron accumulation that is visualized by magnetic resonance imaging (MRI) at the substantia nigra was associated with the disease severity in Parkinson’s disease patients, suggesting the therapeutic potential of iron-chelating therapy [102].

### 3.2. The Mechanisms of Ferroptosis

Ferroptosis is initiated by specific enzymatic pathways or iron-catalyzed free radical chain reactions that are suppressed by direct ROS scavenging, such as vitamin E, coenzyme Q_10_ (CoQ_10_), and ferrostatin-1, or the supplementation of GSH sources, such as NAC and cystine, iron-chelating, such as DFO, or the inhibition of glutaminolysis, lipoxygenases (LOXs), acyl-CoA synthetase long chain family member 4 (ACSL4), or lysophosphatidylcholine acyltransferase 3 (LPCAT3) [98]. Conversely, ferroptosis is induced through the inhibition of a cystine/glutamate antiporter (SLC3A2/SLC7A11 heterodimer), also known as xCT with erastin or glutamate, depleting GSH with BSO, or inhibiting glutathione peroxidase 4 (GPX4) with (1S,3R)-RSL3 (RSL3) [98]. Among the eight distinct isoforms of GPXs, only GPX4 catalyzes detoxifying phospholipid hydroperoxide (PL-OOH) at the cellular membrane, which inhibits membrane instability and permeabilization as follows: PL-OOH + 2 GSH → PL-OH (phospholipid alcohol) + GSSG + H_2_O, while the other GPXs reduce H_2_O_2_ and small fatty acid hydroperoxides, including soluble free fatty acid peroxides [104,105,106]. Furthermore, *L*-glutamine (Gln), which is hydrolyzed by glutaminase to Glu, induced ferroptosis that was ameliorated by inhibiting transaminase from converting Glu to α-ketoglutarate [107]. Indeed, α-ketoglutarate, succinate, fumarate, and malate promoted cystine-deprivation-induced ferroptosis via the hyperpolarization of the mitochondrial membrane potential [108], indicating that the activated TCA cycle causes ferroptosis by the means of dysregulated mitochondrial respiration when fueled by glutaminolysis. In addition to these metabolites, the basal NADPH level was correlated with sensitivity to ferroptosis inducers [109]. NADPH contributes to the following reactions: GSSG+ 2 NADPH → 2 GSH + 2 NADP^+^ by GSH reductase, CoQ_10_H^•^/α-Toc + NADPH → CoQ_10_H_2_/α-Toc + NADP^+^ by ferroptosis suppressor protein 1 (FSP1), and BH_2_ (dihydrobiopterin) + 2 NADPH → BH_4_ + 2 NADP^+^ by dihydrofolate reductase (DHFR) in combination with guanosine triphosphate cyclohydrolase 1 (GCH1), the rate-limiting synthetic enzyme for BH_4_ that scavenges ROS and suppresses ferroptosis in a GPX4-independent manner [93]. Taken together, the disrupted homeostasis of iron, amino acids, thiols, and PUFAs with oxygenating machinery is critical in ferroptotic cascades (Figure 4B).

### 3.3. Ferroptosis in the Kidney and RCC

The cKO of *Gpx4*, which is expressed at higher levels in the proximal tubular cells than it is in the glomeruli, caused AKI with increased phosphatidylcholine (PC)-, phsphatidylethanolamine (PE)-, and cardiolipin (CL)-esterified linoleic acid (LA, C18:2), arachidonic acid (AA, C20:4), and docosahexaenoic acid (DHA, C22:6) oxidations, while *Gpx4* KO mice showed embryonic lethality [105,110]. Other studies focusing on *Gpx4* cKO mice disclosed that PE-AA and PE-adrenoyl (AdA, C22:4) acyls transduce ferroptotic death signaling in the MEFs and kidney [103]. In AKIs, renal tubular death that is mediated by caspases-mediated apoptosis and inflammasome-mediated pyroptosis is detected less frequently than necrosis and ferroptosis [111]. The amelioration of renal IRI by the ferroptosis inhibitor was enhanced by the combination of the necrosis inhibitor that suppressed RIPK, indicating the interaction of these signaling pathways [112], while the replacement of the active selenocysteine in Gpx4 did not become sensitized to cisplatin-induced AKI, indicating that impaired Gpx4 did not aggravate all of the AKIs [113].

In the ccRCC cell lines, erastin and BSO inhibited cellular growth, indicating the importance of GSH biosynthesis and the GPX-dependent detoxifying pathway [114]. Indeed, the tumoricidal activity of erastin was associated with the SLC7A11 expression levels [115]. Furthermore, in HLRCC-patient-derived RCC cell lines harboring the *FH* mutation, the increased proliferation with resistance to cystine-starvation-induced ferroptosis was canceled by the forced expression of Wt FH [108], suggesting that the induction of ferroptosis contributes to RCC suppression.

### 3.4. Induction of Oxidized Lipids and RCC by Fe-NTA

Fe-NTA generated a wide range of C_2–12_ aldehydes and C_7–12_ acyloins, metabolites of saturated aldehydes in the renal and hepatic tissues of male Wistar rats [116]. Among these oxidized lipids, a single injection of Fe-NTA elevated 4-hydroxy-2-nonenal (HNE) at the highest fold change (27.3-fold) and malondialdehyde (MDA), the most abundant aldehyde in the kidney [116]. A single Fe-NTA injection also decreased PUFAs, such as AA and DHA to 70–80%, while linoleic acid was not significantly oxidized in male Wistar rats [117]. In line with previous reports indicating that the supplementation of AA enhanced RSL-3-induced ferroptosis [103], the supplementation of dietary DHA enhanced Fe-NTA-induced renal oxidative injury, which could be restored by α-Toc supplementation in mice [118]. These results indicate that dietary lipids modified Fe-NTA-induced oxidative stress (Figure 4A). In addition to elevated lipid peroxidations, Fe-NTA decreased ferroptosis-suppressive substrates, such as GSH and NADPH [119]. Simultaneously, Fe-NTA activated Nrf2, which suppresses ferroptosis by inducing detoxifying enzymes [104]; thus, the final redox balance in the microenvironments may affect the fates of cells. Furthermore, the *Gpx4* heteroKO mice prolonged their life spans by increasing the susceptibility to cell death compared to Wt mice [120]. Indeed, the A/J strain, which develops higher Fe-NTA-induced RCC, yielded lower oxidative DNA damage, lipid peroxidation, and ferroptosis than the C57/BL6 strain, indicating the role of ferroptosis in cancer prevention by eliminating mutation-prone cells [63].

An epidemiological study to assess the risk of RCC onset provided no clear association between the dietary intake of ascorbate or vitamin E and RCC risk [121]; recently, an umbrella review of meta-analyses focusing on the prevention of RCC in dietary compounds indicated no high-quality evidence or moderate-quality evidence of vitamin C intake and low-quality evidence of α-Toc and calcium intake [122], indicating the difficulty of translating the preventive clinical measures from basic research. In human trials for chemoprevention by antioxidative compounds on asbestos-exposed workers or smokers, the number of lung cancer patients increased after supplemental β-carotene with retinol, which is presumed to have a toxic pro-oxidant effect that contaminates breakdown products or affects uptake modulation for other carotenoids [20]. In addition to these factors, this tumor promotional effect of β-carotene may be initiated by the escape of mutated cells from ferroptosis (Figure 4B).

## 4. Conclusions

In mammals, the tight regulation of intestinal iron absorption from the diet inhibits iron-mediated tissue injury. Meanwhile, in experimental models, the intraperitoneal injection of Fe-NTA initiates oxidative injury by the GSH-cycle-dependent reduction of ferric to ferrous ion. Furthermore, the repeated injection of Fe-NTA causes the inactivation of *Cdkn2a/2b* in renal proximal tubules that finally develop renal carcinogenesis in rodents. In contrast to iron-induced carcinogenesis, iron-dependent cell death is established as ferroptosis. Ferroptosis forms a “wave of death” in renal tubular cells, such as free-radical-initiated propagative chain reactions. Indeed, a multi-target drug combining iron chelation, ROS-scavenging, and anti-inflammatory effects is proposed to restore ischemic injury by terminating ferroptosis [102]. In addition, the ferroptosis induction that is based on the understanding of redox biology is an emerging and promising modality for cancer therapy. Further study is warranted to explore the role of ferroptosis in clinical medicine.

## Figures and Tables

**Table 1:** Inherited renal cancer syndromes with predisposing genes: the chromosomal location, predominant histopathology, and activated signaling pathway.

Syndrome	Predisposing Gene	Chromosomal Location	Predominant Histopathology	Activated Signaling Pathway
von Hippel–Lindau (VHL) disease	*VHL*	3p25	Clear cell	HIF pathway
Hereditary papillary renal cell carcinoma	*MET*	7q31	Papillary (type 1)	MET kinase pathway
Birt–Hogg–Dubé syndrome	*FLCN* (*Folliculin*)	17p11	Chromophobe, hybrid oncocytic, and clear cell	mTOR pathway
Hereditary leiomyomatosis and renal cell carcinoma (HLRCC)	*FH* (*Fumarate hydratase*)	1q43	Papillary (type 2)	HIF pathway, NRF2 pathway, and TCA cycle pathway
Succinate dehydrogenase-deficient renal cell carcinoma (SDH-deficient RCC)	*SDHB* *SDHC* *SDHD*	1p36 1q23 11q23	Oncocytic is most common and clear cell is less frequent	HIF pathway and TCA cycle pathway
Tuberous sclerosis complex (TSC)	*TSC1* *TSC2*	9q34 16p13	Heterogeneous; chromophobe, oncocytic, and cystic	mTOR pathway
Cowden syndrome	*PTEN*	10q23	Chromophobe	PI3/AKT/mTOR pathway
Inherited RCC	*CDKN2B*	9q21	Clear cell	Cell cycle regulation
BAP1 tumor predisposition syndrome	*BAP1* (*BRCA1-associated Protein 1*)	3p21	^#^ Clear cell or eosinophilic, high-grade nuclei	Dysregulated BAP1 transcriptional activity, including metabolic reprogramming
MiTF-associated cancer syndrome	*MiTF* (*Microphthalmia-associated transcription factor*)	3p14	^##^ Papillary (type 1) and/or clear cell or demonstrate TFE3-positive Xp11 translocation RCC	Dysregulated MiTF transcriptional activity, including mTOR pathway

^#^ In sporadic *BAP1*-mutated RCC, its histopathology sometimes overlaps with Xp11 translocation RCC, which is characterized by papillary and epithelioid growth [89]. ^##^ Due to the small number of reports, the histological subtype of RCC that is associated with the *MiTF* mutation has shown variable characteristics [88].

## Abbreviations

AKI	acute kidney injury
BN/F344	Brown Norway/Fischer344
DFO	deferoxamine
DMT1	divalent metal transporter 1
Fe-NTA	ferric nitrilotriacetate
GSH	glutathione
GPX4	glutathione peroxidase 4
HCC	hepatocellular carcinoma
HD	homozygous deletion
IRE	iron-responsive element
IRI	ischemia-reperfusion injury
IRP	iron-regulatory protein
LOH	loss of heterozygosity
MM	malignant mesothelioma
RCC	renal cell carcinoma
ROS	reactive oxygen species
Wt	wild-type
